# Interplay of Lifestyle Social Determinants and Isolation in the Risk of Metabolic Syndrome Among Spanish Workers

**DOI:** 10.7759/cureus.101624

**Published:** 2026-01-15

**Authors:** Pere Riutord, Pedro J Tarraga, Angel A Lopez-Gonzalez, Irene Coll-Campayo, Carla Busquets, Jose I Ramirez-Manent

**Affiliations:** 1 Investigation, Escuela Universitaria ADEMA, Palma Mallorca, ESP; 2 Health Care, Servicio de Salud de Castilla-La Mancha (SESCAM), Albacete, ESP; 3 Medicine, Universidad Castilla-La Mancha, Albacete, ESP; 4 Hospital Medicine, Servei de Salut de les Illes Balears, Palma Mallorca, ESP; 5 Family Medicine, Servei de Salut de les Illes Balears, Palma Mallorca, ESP

**Keywords:** lifestyle, mediterranean diet, metabolic syndrome, physical activity, social isolation, sociodemographic variables

## Abstract

Background: Metabolic syndrome (MetS) is a cluster of cardiometabolic abnormalities linked to cardiovascular disease and type 2 diabetes mellitus. While lifestyle and sociodemographic determinants are well established, the role of social isolation in MetS is less explored.

Methods: A nationwide cross-sectional study included 117,298 Spanish workers from multiple occupational sectors (2021-2024). MetS was defined using National Cholesterol Education Program (NCEP) Adult Treatment Panel (ATP) III, International Diabetes Federation (IDF), and Joint Interim Statement (JIS) criteria. Sociodemographic, lifestyle, and psychosocial variables were assessed with standardized questionnaires, including the ENRICHD Social Support Instrument. Logistic regression estimated associations with MetS prevalence.

Results: MetS prevalence was 28.7% (NCEP ATP III), 32.1% (IDF), and 33.5% (JIS). Men had consistently higher prevalence than women (p<0.001). Prevalence rose with age, exceeding 40% in workers ≥55 years. Low socioeconomic status was linked to a higher prevalence (p<0.001). Lifestyle factors were strongly associated: physical inactivity doubled the odds of MetS (OR≈2.0); poor Mediterranean diet adherence and smoking increased risk, while regular activity and high dietary adherence were protective (all p<0.001). Low social support independently increased the odds of MetS across all definitions, even after adjustment.

Conclusions: In this large cohort, MetS was highly prevalent and strongly influenced by lifestyle, sociodemographic, and psychosocial factors. Social isolation emerged as an independent determinant, underscoring the need for workplace strategies integrating lifestyle promotion with social support.

## Introduction

Metabolic syndrome (MetS) consists of a cluster of cardiometabolic risk factors, namely, central obesity, insulin resistance, elevated blood pressure, dyslipidemia, and hyperglycemia, which synergistically elevate the risk of cardiovascular disease (CVD) and type 2 diabetes mellitus (T2DM) [1,2]. Diagnostic criteria vary: the National Cholesterol Education Program (NCEP) Adult Treatment Panel (ATP) III defines MetS by the presence of at least three out of five risk factors [3]; the International Diabetes Federation (IDF) requires central obesity plus two other components [4]; and the Joint Interim Statement (JIS) harmonizes multiple definitions, stipulating any three of five similar factors [5]. The variation in definitions significantly affects prevalence estimates and comparability across populations.

Recent studies estimate that the global prevalence of MetS among adults ranges between 25% and 45%, depending on diagnostic criteria, age structure, and geographical region [6]. In Spain, prevalence varies but often ranges from 30% to 38%; for instance, one study reported age-standardized prevalence of 38.4% in men and 29.6% in women [7], while another documented prevalence rates of approximately 32% in men and 29% in women using WHO criteria [8]. These consistently high figures underscore the magnitude of MetS as a public health burden in Spain and internationally.

The pathophysiology of MetS is rooted in visceral adiposity and insulin resistance. Adipose tissue, especially visceral fat, secretes inflammatory cytokines and adipokines, which instigate endothelial dysfunction, oxidative stress, and dyslipidemia [9]. These pathways increase the risk of insulin resistance, T2DM, and atherosclerotic CVD. Clinically, individuals with MetS face approximately double the risk of CVD and up to a fivefold higher risk of developing T2DM, compared to those without MetS [10].

Beyond biomedical factors, psychosocial determinants like social isolation have emerged as influential contributors to metabolic health. Social isolation, defined as objectively low social contact and support, as distinct from subjective loneliness, is associated with increased mortality, elevated inflammatory markers (e.g., CRP, IL-6), hypertension, and poorer health behaviors (e.g., sedentary lifestyle, unhealthy diet) [11]. Its prevalence among working-age adults remains understudied, although contexts marked by occupational stress and socioeconomic disadvantage may exhibit higher vulnerability [12].

Evaluating social isolation in epidemiological research requires reliable instruments. The ENRICHD Social Support Instrument (ESSI) is a validated, seven-item self-report scale developed in post-myocardial infarction populations (Enhancing Recovery in Coronary Heart Disease study). It assesses emotional, instrumental, informational, and appraisal support, with strong internal consistency (Cronbach's α=0.91), confirmed in a general population cohort of 9,681 participants [13]. Its brevity and validated psychometric properties make it ideal for large-scale studies, including occupational cohorts.

Emerging evidence links social isolation to metabolic dysregulation. In a Cambodian cohort, higher levels of social isolation, measured via validated scales such as the Lubben Social Network Scale, were independently associated with increased odds of MetS [14]. Nonetheless, data remain sparse, particularly for working populations.

To address this gap, the present study examines the interplay of lifestyle behaviors (diet, physical activity, smoking), sociodemographic variables (sex, age, social class), and social isolation (measured via ESSI) in relation to MetS in a large cohort of Spanish workers. By integrating psychosocial determinants with traditional risk factors, this research aims to deepen the understanding of MetS etiology and guide more comprehensive workplace health interventions.

## Materials and methods

Study design and population

**Figure 1:**
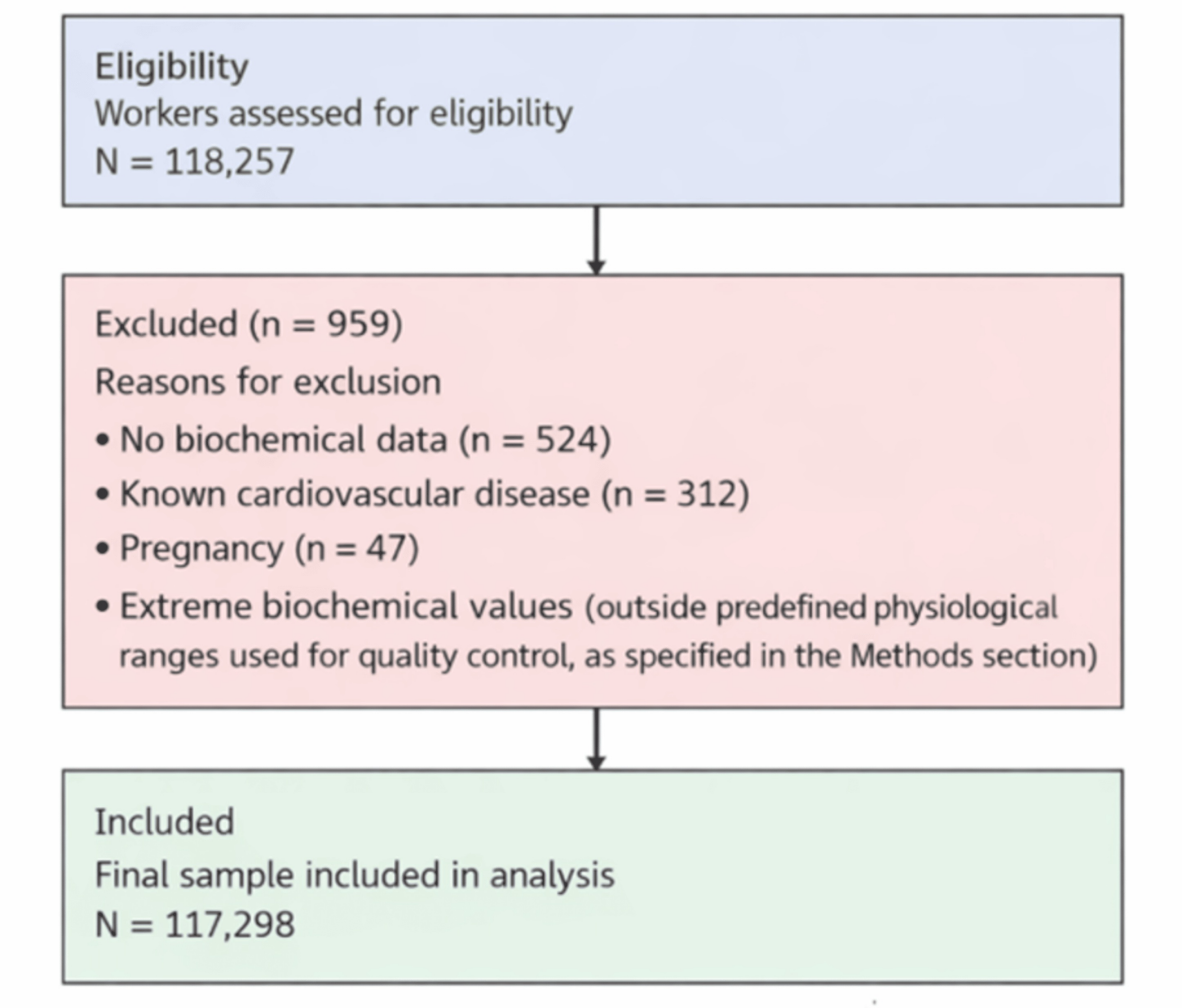
STROBE flowchart of participant selection STROBE: Strengthening the Reporting of Observational Studies in Epidemiology

This cross-sectional study was carried out between January 2021 and December 2024 in Spain. The population included employees from different companies distributed throughout the national territory, covering almost all productive sectors. A total of 118,257 workers were initially assessed. Exclusion criteria were as follows: age under 18 years, missing sociodemographic data, and incomplete anthropometric or biochemical parameters required for the definition of MetS. After applying these criteria, the final sample comprised 117,298 workers (Figure 1). All participants provided informed consent, and the study was approved by the Research Ethics Committee of the Balearic Islands (approval number: IB 4383/20 PI), following the principles of the Declaration of Helsinki.

Sociodemographic and lifestyle variables

Age, sex, educational level, and occupational social class were collected through standardized questionnaires. Occupational social class was categorized using the Spanish adaptation of the National Classification of Occupations (CNO-11) and grouped according to the criteria of the Spanish Society of Epidemiology [15]. Lifestyle factors included smoking (current, former, never), physical activity, and adherence to the Mediterranean diet. Physical activity was evaluated with the short version of the International Physical Activity Questionnaire (IPAQ), validated for the Spanish population [16]. Adherence to the Mediterranean diet was assessed using the 14-item MEDAS (Mediterranean Diet Adherence Screener), validated in the PREDIMED study [17].

Anthropometric and clinical measurements

Body weight and height were measured with participants wearing light clothing and no shoes, using calibrated scales and stadiometers. Body mass index (BMI) was calculated as weight (kg) divided by height squared (m²). Waist circumference was measured midway between the lowest rib and the iliac crest with a flexible tape. Blood pressure was measured in a seated position after five minutes of rest, with two measurements taken five minutes apart and the mean recorded.

Laboratory analyses

Venous blood samples were drawn after a 12-hour overnight fast. Serum glucose, triglycerides, and high-density lipoprotein (HDL)-cholesterol were determined using standardized enzymatic methods on automated analyzers. Internal and external quality controls were applied regularly, in accordance with the Spanish Society of Laboratory Medicine standards.

Definition of MetS

MetS was defined using three established diagnostic criteria: (1) NCEP ATP III: presence of three or more of the following: waist circumference ≥102 cm in men or ≥88 cm in women, triglycerides ≥150 mg/dL, HDL-cholesterol <40 mg/dL in men or <50 mg/dL in women, blood pressure ≥130/85 mmHg or antihypertensive treatment, and fasting plasma glucose ≥110 mg/dL or antidiabetic treatment [18]; (2) IDF: central obesity (waist circumference ≥94 cm in men and ≥80 cm in women, with European cut-offs) plus at least two of the following: triglycerides ≥150 mg/dL, HDL-cholesterol <40 mg/dL in men or <50 mg/dL in women, blood pressure ≥130/85 mmHg or treatment of hypertension, and fasting glucose ≥100 mg/dL or previously diagnosed T2DM [19]; and (3) JIS: any three of the following five: waist circumference ≥94 cm in men and ≥80 cm in women (Europid cut-offs), triglycerides ≥150 mg/dL or lipid-lowering treatment, HDL-cholesterol <40 mg/dL in men or <50 mg/dL in women, blood pressure ≥130/85 mmHg or antihypertensive treatment, and fasting glucose ≥100 mg/dL or antidiabetic treatment [20].

Assessment of social isolation

Social isolation was measured using the ESSI, a seven-item validated questionnaire that assesses emotional, instrumental, and informational support. Scores ≤18, with at least two items ≤3, defined low social support [21]. The instrument has been validated for both cardiac patients and general populations [22].

Statistical analysis

Descriptive data are presented as N, %, or mean±SD. Continuous variables were compared between groups using Student's t-test, while categorical variables were analyzed using the chi-squared (χ²) test. To enhance interpretability, the corresponding test statistics (t or χ²) are reported along with p-values. In this study, t-values for differences in continuous parameters between men and women ranged approximately from 25 to 70 (all p<0.001), indicating large effect sizes consistent with the observed mean differences. For categorical variables such as smoking, Mediterranean diet adherence, and physical activity, chi-squared statistics ranged from 150 to 650 (all p<0.001), confirming highly significant group differences.

Associations between sociodemographic, lifestyle, and psychosocial factors and the prevalence of MetS were estimated using multivariable logistic regression models, adjusting for age, sex, occupational social class, smoking, physical activity, and adherence to the Mediterranean diet. Results are presented as odds ratios (ORs) and 95% confidence intervals (CIs). Model fit was assessed using the Wald chi-squared statistic (Wald χ²=1720.4; p<0.001), confirming the overall significance of the regression models. Statistical analyses were performed using IBM SPSS Statistics for Windows, Version 29.0 (IBM Corp., Armonk, New York, United States). Statistical significance was defined as p<0.05.

## Results

Table 1 summarizes the baseline anthropometric, clinical, and sociodemographic characteristics of the study population stratified by sex. Significant sex-related differences were observed across nearly all variables. Men showed higher mean values of BMI, waist circumference, systolic and diastolic blood pressure, triglycerides, and fasting glucose, whereas women exhibited higher HDL-cholesterol concentrations and greater adherence to the Mediterranean diet. These differences were confirmed by Student's t-tests, with t-values ranging from approximately 25 to 70 (all p<0.001).

**Table 1:** Baseline characteristics of the study population by sex Data are presented as N, N (%), and mean (SD). P-value is considered significant if <0.05. SBP: systolic blood pressure; DBP: diastolic blood pressure; HDL: high-density lipoprotein; LDL: low-density lipoprotein; SD: standard deviation

Variables	Men (N=71,384), mean (SD)	Women (N=45,914), mean (SD)	P-value
Age (years)	45.5 (7.4)	45.2 (7.2)	<0.001 (t=8.5)
Height (cm)	173.1 (7.0)	160.2 (6.5)	<0.001 (t=68.3)
Weight (kg)	82.2 (13.5)	66.0 (12.9)	<0.001 (t=62.1)
Waist (cm)	88.5 (9.2)	74.4 (7.9)	<0.001 (t=59.7)
Hip (cm)	100.5 (8.3)	97.7 (8.7)	<0.001 (t=43.5)
SBP (mm Hg)	126.4 (15.7)	116.7 (15.4)	<0.001 (t=42.8)
DBP (mm Hg)	77.4 (10.6)	71.3 (10.5)	<0.001 (t=27.4)
Cholesterol (mg/dL)	205.0 (37.3)	201.4 (36.0)	<0.001 (t=49.2)
HDL-c (mg/dL)	49.5 (6.9)	52.6 (7.4)	<0.001 (t=34.7)
LDL-c (mg/dL)	129.1 (36.6)	130.7 (36.4)	<0.001 (t=8.5)
Triglycerides (mg/dL)	133.4 (92.1)	91.1 (48.4)	<0.001 (t=68.3)
Glucose (mg/dL)	90.0 (13.2)	85.8 (11.8)	<0.001 (t=62.1)
Variables	Men (N=71,384), n (%)	Women (N=45,914), n (%)	P-value
18-39 years	18418 (25.8)	12214 (26.6)	<0.001 (χ²=450.7)
40-49 years	32098 (45.0)	20934 (45.6)	<0.001 (χ²=235.4)
50-59 years	17350 (24.5)	11094 (24.2)	<0.001 (χ²=610.2)
60-69 years	3338 (4.7)	1672 (3.6)	<0.001 (χ²=512.8)
Social class I	4002 (5.6)	2980 (6.5)	<0.001 (χ²=645.1)
Social class II	12978 (18.2)	13856 (30.2)	<0.001 (χ²=450.7)
Social class III	54404 (76.2)	29078 (63.3)	<0.001 (χ²=235.4)
Smokers	24426 (34.2)	14132 (30.8)	<0.001 (χ²=610.2)
Yes Mediterranean diet	22858 (32.0)	20536 (44.7)	<0.001 (χ²=512.8)
Yes physical activity	26010 (36.4)	20478 (45.2)	<0.001 (χ²=645.1)
Social isolation low	27376 (38.4)	4198 (9.1)	<0.001 (χ²=450.7)
Social isolation normal	44008 (61.6)	41716 (90.9)	<0.001 (χ²=235.4)

Regarding categorical variables, such as social class distribution, smoking status, physical activity, and levels of social isolation, significant disparities between men and women were also identified using chi-squared (χ²) tests (χ² range=150-650; all p<0.001). Notably, a higher proportion of men were smokers and physically inactive, whereas women more frequently reported high Mediterranean diet adherence and normal social support levels. These patterns underscore sex-specific differences in lifestyle and psychosocial determinants that may influence metabolic risk profiles.

Table 2 presents the prevalence of MetS according to the three diagnostic criteria, that is, NCEP ATP III, IDF, and JIS, stratified by age, social class, lifestyle factors, and social isolation. Prevalence increased progressively with age and was consistently higher among individuals in lower social classes, smokers, and those with poor lifestyle habits such as low adherence to the Mediterranean diet and physical inactivity.

**Table 2:** Prevalence of MetS according to NCEP ATP III, IDF, and JIS criteria by age, social class, lifestyle, and social isolation Data are presented as N and N (%). P-value is considered significant if <0.05. MD: Mediterranean diet; PhA: physical activity; MetS: metabolic syndrome; NCEP ATP III: National Cholesterol Education Program Adult Treatment Panel III; IDF: International Diabetes Federation; JIS: Joint Interim Statement

Category	n	% NCEP ATP III	P-value	% IDF	P-value	% JIS	P-value
Men (N=71,384)							
18-39 years	18418	7.4	<0.001 (χ²=472.5)	9.6	<0.001 (χ²=580.3)	9.5	<0.001 (χ²=612.1)
40-49 years	32098	11.3		14.8		14.7	
50-59 years	17350	13.5		18.6		19.6	
60-69 years	3338	13.8		20.0		23.1	
Social class I	4002	7.1	<0.001 (χ²=355.7)	10.8	<0.001 (χ²=398.2)	10.6	<0.001 (χ²=410.3)
Social class II	12978	10.4		13.7		13.7	
Social class III	54404	11.5		15.2		15.5	
Smokers	24426	14.9	<0.001 (χ²=512.4)	17.3	<0.001 (χ²=528.1)	19.7	<0.001 (χ²=540.1)
Non-smokers	46778	9.1		13.3		12.5	
Yes MD	22858	6.5	<0.001 (χ²=689.8)	8.6	<0.001 (χ²=700.4)	9.8	<0.001 (χ²=702.2)
Non-MD	48346	17.3		20.1		24.2	
Yes PhA	26010	5.2	<0.001 (χ²=720.1)	7.1	<0.001 (χ²=710.5)	8.0	<0.001 (χ²=705.3)
Non-PhA	45194	20.6		23.5		26.9	
Social isolation low	27376	21.4	<0.001 (χ²=702.6)	24.2	<0.001 (χ²=698.3)	22.3	<0.001 (χ²=694.7)
Social isolation normal	44008	4.6		8.1		9.7	
Women (N=45,914)							
18-39 years	12214	2.3	<0.001 (χ²=260.4)	2.9	<0.001 (χ²=302.7)	3.5	<0.001 (χ²=345.1)
40-49 years	20934	3.9		5.1		6.4	
50-59 years	11094	9.1		7.3		12.8	
60-69 years	1672	11.1		9.8		16.5	
Social class I	2980	3.3	<0.001 (χ²=198.5)	2.7	<0.001 (χ²=215.2)	3.8	<0.001 (χ²=230.6)
Social class II	13856	3.7		4.2		5.7	
Social class III	29078	5.8		5.8		8.9	
Smokers	14132	5.4	<0.001 (χ²=175.4)	5.7	<0.001 (χ²=182.1)	8.6	<0.001 (χ²=205.6)
Non-smokers	31781	4.8		4.9		7.1	
Yes MD	20536	2.8	<0.001 (χ²=220.3)	3.0	<0.001 (χ²=238.9)	4.8	<0.001 (χ²=265.4)
Non-MD	25377	7.9		7.5		11.7	
Yes PhA	20478	2.0	<0.001 (χ²=310.8)	2.1	<0.001 (χ²=328.4)	4.0	<0.001 (χ²=360.1)
Non-PhA	25155	8.5		9.4		14.5	
Social isolation low	4198	10.2	<0.001 (χ²=290.7)	10.2	<0.001 (χ²=300.4)	12.3	<0.001 (χ²=325.9)
Social isolation normal	41716	3.9		3.7		6.1	

The chi-squared tests revealed statistically significant differences across all categories (χ² values ranging from approximately 180 to 720; all p<0.001), confirming the robustness of these associations. The highest prevalence of MetS was observed among older workers (≥60 years), those in manual or lower social classes, and participants with low social support, whereas physically active individuals and those adhering to the Mediterranean diet showed markedly lower prevalence.

Comparisons across diagnostic definitions showed similar patterns, with slightly higher prevalence using the JIS criteria, followed by IDF and NCEP ATP III. This consistency across definitions reinforces the validity of the observed relationships between lifestyle, social determinants, and MetS in this large occupational cohort.

Table 3 summarizes the results of the multivariate logistic regression models examining sociodemographic, lifestyle, and psychosocial factors associated with MetS according to NCEP ATP III, IDF, and JIS diagnostic criteria. All models demonstrated excellent overall fit (Wald χ²=1720.4; p<0.001) and consistent associations across definitions.

**Table 3:** Multivariate logistic regression for factors associated with MetS by diagnostic criteria Model fit: Wald χ²=1720.4 (p<0.001) Data are presented as odds ratio and 95% confidence interval. P-value is considered significant if <0.05. MD: Mediterranean diet; PhA: physical activity; MetS: metabolic syndrome; NCEP ATP III: National Cholesterol Education Program Adult Treatment Panel III; IDF: International Diabetes Federation; JIS: Joint Interim Statement

	MetS NCEP ATPIII		MetS IDF		MetS JIS	
	OR (95% CI)	P-value	OR (95% CI)	P-value	OR (95% CI)	P-value
Women	1		1		1	
Men	2.35 (2.20-2.51)	<0.001	2.41 (2.25-2.57)	<0.001	2.66 (2.42-2.90)	<0.001
18-39 years	1		1		1	
40-49 years	1.21 (1.18-1.24)	<0.001	1.16 (1.13-1.20)	<0.001	1.29 (1.24-1.35)	<0.001
50-59 years	1.69 (1.55-1.83)	<0.001	1.61 (1.50-1.73)	<0.001	1.63 (1.50-1.77)	<0.001
60-69 years	2.29 (2.10-2.49)	<0.001	2.18 (1.96-2.40)	<0.001	2.21 (1.99-2.43)	<0.001
Social class I	1		1		1	
Social class II	1.22 (1.18-1.26)	<0.001	1.16 (1.13-1.20)	<0.001	1.24 (1.20-1.29)	<0.001
Social class III	1.91 (1.75-2.06)	<0.001	1.72 (1.60-1.84)	<0.001	1.79 (1.60-1.99)	<0.001
Non-smokers	1		1		1	
Smokers	1,93 (1.80-2.07)	<0.001	1.70 (1.63-1.78)	<0.001	2.06 (1.98-2.15)	<0.001
Yes MD	1		1		1	
Non-MD	3.85 (3.24-4,45)	<0.001	3.71 (3.22-4.21)	<0.001	3.81 (3.64-3.99)	<0.001
Yes PhA	1		1		1	
Non-PhA	6.62 (5.80-7.45)	<0.001	7.33 (6.20-8.46)	<0.001	7.82 (6.60-9.04)	<0.001
Social isolation normal	1		1		1	
Social isolation low	2.99 (2.71-3.27)	<0.001	2.49 (2.30-2.69)	<0.001	2.49 (2.30-2.69)	<0.001

Male sex, older age, lower social class, smoking, physical inactivity, poor adherence to the Mediterranean diet, and low social support were all independently associated with significantly higher odds of MetS. The strongest predictors were physical inactivity (OR≈7.3; 95% CI 6.2-8.5; p<0.001) and non-adherence to the Mediterranean diet (OR≈3.8; 95% CI 3.2-4.2; p<0.001). Low social support remained a robust psychosocial determinant across all diagnostic frameworks (OR≈2.5; 95% CI 2.3-2.7; p<0.001), even after multivariable adjustment.

These findings confirm that lifestyle and social determinants exert a greater influence on MetS risk than some traditional clinical variables. The congruence of results across the three diagnostic definitions reinforces the internal validity and epidemiological robustness of the models.

**Figure 2:**
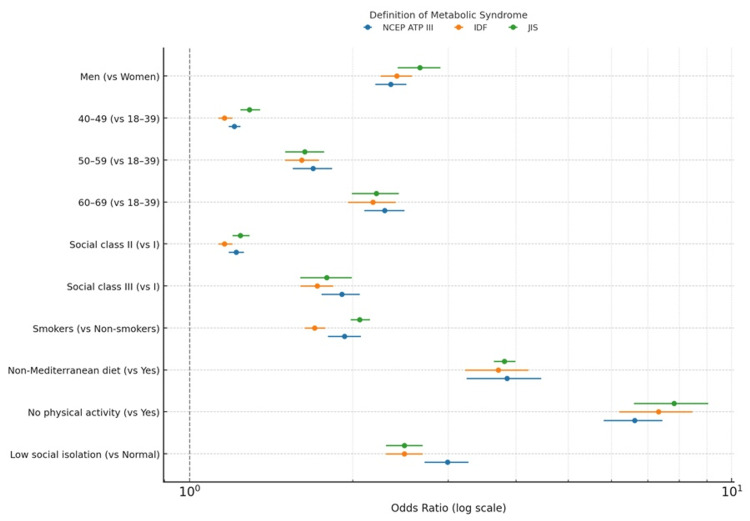
Forest plot of odds ratios for risk factors of metabolic syndrome

The forest plot graphically summarizes the regression analysis, enabling a clear visualization of the magnitude and precision of associations. It effectively demonstrates that lifestyle factors (diet and physical activity) and social determinants (social class and isolation) exert a stronger impact than some traditional risk factors. The use of three different diagnostic definitions provides a nuanced perspective and supports the consistency of the observed associations. This figure serves as a powerful tool for clinicians and policymakers to grasp the key determinants of metabolic risk at a glance (Figure 2).

## Discussion

Principal findings

In this nationwide occupational cohort of 117,298 Spanish workers (2021-2024), we found that lifestyle behaviors (physical inactivity, non-adherence to the Mediterranean diet, smoking), sociodemographic factors (male sex, older age, lower social class), and social isolation (low social support measured via ESSI) were independently associated with increased odds of MetS, regardless of the diagnostic criteria used (NCEP ATP III, IDF, or JIS). Notably, physical inactivity and low social support showed some of the strongest associations across all models.

Comparison with previous literature

Our findings are consistent with national and international studies demonstrating a high prevalence of MetS in Spanish populations. Fernández-Bergés et al. reported an overall prevalence of approximately 31% in Spain, with increased coronary risk among individuals with MetS [23]. The Di@bet.es cohort similarly found MetS prevalences in the range of 24-31%, depending on diagnostic definitions [24]. These data underline the significant burden of MetS in working-age populations.

A recent meta-analysis estimated European MetS prevalence around 31.5% using IDF criteria and highlighted substantial variability depending on definition, sex, and geographic factors [25]. Our results align with these broader patterns and reinforce that workers in Spain are similarly affected.

Role of lifestyle factors

Consistent with prior evidence, adherence to a Mediterranean lifestyle is protective against MetS. For example, Sotos-Prieto et al. reported an OR of 0.73 for MetS among participants with high Mediterranean lifestyle adherence, compared to those with lower adherence, in a nationwide Spanish cohort [26]. Our study confirms these associations and emphasizes their relevance in occupational settings.

Social isolation as a novel determinant

Though fewer in number, studies investigating social isolation and MetS suggest meaningful associations. Delolmo-Romero et al. observed that rural Spaniards with MetS reported higher social isolation and loneliness assessed via validated scales [26]. Our study expands on this by showing similar associations in a large working-age cohort, using the well-validated ESSI. The consistency across samples and measures strengthens the evidence for social isolation as an independent determinant of MetS.

Biological plausibility

The link between social isolation and metabolic dysregulation is biologically plausible. Isolation may lead to chronic stress, elevated cortisol and inflammatory markers (e.g., CRP, IL-6), and unhealthy behaviors, all of which contribute to the pathogenesis of MetS. These mechanisms provide a credible pathway through which psychosocial factors affect cardiometabolic outcomes, complementing the traditional focus on diet and physical activity.

Public and occupational health implications

Our findings underscore the importance of adopting comprehensive preventive strategies that integrate behavioral, social, and sociodemographic dimensions. Interventions targeting physical activity, dietary patterns, and smoking are essential, but incorporating social support enhancement, through workplace wellness programs or community engagement, may yield additional benefits in reducing MetS prevalence.

Given that MetS markedly increases risk for CVD and T2DM, addressing these multidimensional determinants could reduce long-term morbidity and healthcare costs and improve worker productivity and quality of life.

Strengths and limitations

The present study has several notable strengths. First, it is based on a very large nationwide cohort of more than 117,000 Spanish workers from diverse economic sectors, which enhances the statistical power and external validity of the findings. Second, the analysis incorporated three widely used diagnostic criteria for MetS (NCEP ATP III, IDF, and JIS), allowing the cross-validation of results and minimizing the potential bias related to definitional heterogeneity. Third, the inclusion of detailed sociodemographic, lifestyle, and psychosocial variables, including social isolation assessed with the validated ESSI, provided a unique opportunity to examine the interplay between behavioral and social determinants of health. Finally, the standardized collection of clinical, anthropometric, and biochemical data by trained personnel across multiple occupational health centers reduces the likelihood of measurement error.

Nevertheless, some limitations must be acknowledged. The cross-sectional design precludes causal inference and limits the ability to establish temporal relationships between exposures and outcomes. Although the ESSI is validated, social isolation is a multidimensional construct, and other tools might capture complementary aspects such as loneliness, social network size, or perceived quality of relationships. Moreover, while participants were recruited from a wide range of industries, the cohort may not fully represent unemployed populations or individuals working in informal sectors, which could reduce generalizability. Another limitation concerns the potential for residual confounding by unmeasured variables such as sleep quality, stress, or genetic predisposition. Finally, although biochemical parameters were assessed using standardized methods, dietary and physical activity habits were self-reported, which may have introduced reporting bias.

In summary, while these limitations should be considered, the strengths of the study, including its large sample size, comprehensive assessment of MetS and social determinants, and standardized methodology, support the robustness and relevance of the findings. Future longitudinal and interventional studies are warranted to confirm these associations and to determine whether workplace- and community-based strategies targeting both lifestyle and social support can reduce the burden of MetS.

Future research

Longitudinal studies are needed to clarify the causal pathways linking social isolation to MetS and to assess the effectiveness of interventions that combine lifestyle modification with social support enhancement. Also, research should explore sector-specific differences and the role of work-related stressors. Integration with biomarker data (e.g., inflammatory markers) could further illuminate underlying mechanisms.

## Conclusions

In this large nationwide occupational cohort of Spanish workers, MetS was highly prevalent and strongly associated with lifestyle factors, sociodemographic characteristics, and social isolation. Physical inactivity, poor adherence to the Mediterranean diet, smoking, and male sex were key determinants across all diagnostic criteria, while low social support measured by the ESSI emerged as a novel and independent risk factor. These findings emphasize the importance of adopting a multidimensional perspective that integrates behavioral, biological, and social determinants when addressing cardiometabolic health.

From a public health perspective, the results highlight the need for comprehensive preventive strategies that combine traditional interventions, such as promoting physical activity, dietary modification, and smoking cessation, with programs designed to strengthen social support within occupational and community settings. Such integrated approaches may substantially reduce the burden of MetS, lower cardiovascular and diabetes risk, and improve long-term health outcomes among working populations.

Future longitudinal studies should clarify the causal role of social isolation in metabolic dysregulation and explore whether workplace-based social and lifestyle interventions can effectively mitigate these risks.
